# High‐resolution mapping of 33 years of material stock and population growth in Germany using Earth Observation data

**DOI:** 10.1111/jiec.13343

**Published:** 2022-10-25

**Authors:** Franz Schug, David Frantz, Dominik Wiedenhofer, Helmut Haberl, Doris Virág, Sebastian van der Linden, Patrick Hostert

**Affiliations:** 1 Geography Department, Humboldt‐Universität zu Berlin, Unter den Linden 6, 10099 Berlin, Germany; 2 Integrated Research Institute on Transformations of Human Environment Systems, Humboldt‐Universität zu Berlin, Berlin, Germany; 3 Geoinformatics – Spatial Data Science, Trier University, Trier, Germany; 4 Institute of Social Ecology, University of Natural Resources and Life Sciences, Vienna, Austria; 5 Institute of Geography and Geology, University of Greifswald, Greifswald, Germany

**Keywords:** buildings, CAT transform, industrial ecology, infrastructure, Landsat, socio‐economic metabolism, time series analysis

## Abstract

Global societal material stock in buildings and infrastructure have accumulated rapidly within the last decades, along with population growth. Recently, an approach for nation‐wide mapping of material stock at 10 m spatial resolution, using freely available and globally consistent Earth Observation (EO) imagery, has been introduced as an alternative to cost‐intensive cadastral data or broad‐scale but thematically limited nighttime light‐based mapping. This study assessed the potential of EO data archives to create spatially explicit time series data of material stock dynamics and their relation to population in Germany, at a spatial resolution of 30 m. We used Landsat imagery with a change‐aftereffect‐trend analysis to derive yearly masks of land surface change from 1985 onward. Those served as an input to an annual reverse calculation of six material stock types and building volume‐based annual gridded population, based on maps for 2018. Material stocks and population in Germany grew by 13% and 4%, respectively, showing highly variable spatial patterns. We found a minimum building stock of ca. 180 t/cap across all municipalities and growth processes characterized by sprawl. A rapid growth of stocks per capita occurred in East Germany after the reunification in 1990, with increased building activity but population decline. Possible over‐ or underestimations of stock growth cannot be ruled out due to methodological assumptions, requiring further research.

**Supplementary Information:**

The online version of this article (doi:10.1111/jiec.13343) contains supplementary material, which is available to authorized users.

## INTRODUCTION

In the second half of the 21st century, a strong growth of global human population and economic activity went along with a rapid accumulation of societal material stock (Krausmann et al., 2017). Societal material stock encompasses all materials contained in buildings, infrastructure, and other durable goods. They are considered the basis for human living and well‐being, as they provide key services such as shelter, food, mobility, or health (Haberl et al., 2017; Lanau et al., 2019). Here we use the term “material stock,” which is largely synonymous with concepts such as *in‐use stocks* (Pauliuk & Müller, 2014), *technomass* (Inostroza, 2014), or *manufactured capital* (Weisz et al., 2015). Material stock represents long‐lived resource use, and has an important impact on processes that contribute to unwanted (e.g., climate) change—both immediate, for example, through emissions caused by the extraction and the processing of primary resources (Hertwich, 2021), and indirect, for example, through shaping human behavior (Haberl, Schmid et al., 2021). Similar to global population growth (UNDESA, 2019), material stock is expected to further accumulate across the world (Krausmann et al., 2020; Wiedenhofer et al., 2021). A better understanding of patterns and dynamics of material stock is, thus, required for developing strategies and policies for sustainable resource use (Lanau et al., 2019; Pauliuk & Müller, 2014).

The concept of socio‐economic metabolism (SEM) underpins the need for research on material turnover and stocks (Ayres & Simonis, 1994; Fischer‐Kowalski & Haberl, 1998; Pauliuk & Hertwich, 2015). In this approach, society is conceptualized as a hybrid of material and biophysical entities (Fischer‐Kowalski & Weisz, 1999), whose biophysical basis includes resource extraction and conversion, production and consumption, the accumulation of material stock in buildings, infrastructure and machinery, as well as people and livestock, and the resulting wastes and emissions (Haberl et al., 2019). The spatial distribution of material stock shapes social practices and the systems that provide services to society (Haberl, Wiedenhofer et al., 2021). Material stock patterns are relevant for a number of topics, such as spatial planning, urban sprawl (Barrington‐Leigh & Millard‐Ball, 2015), waste management, urban mining, and circular economy strategies (Haas et al., 2020; Lederer et al., 2021), as well as other environmental concerns linked to stocks, their use, and the impacts on surrounding ecosystems (Elhacham et al., 2020). Material stocks provide key services such as shelter and mobility. Measures of material stocks per capita are increasingly used in socio‐ecological research, for example, as indicator for spatial inequalities in terms of available infrastructures, sustainable policies and practices, resource use efficiency, or socio‐economic trajectories between regions, but also on local levels within countries (Haberl et al., 2019; Haberl, Schmid et al., 2021; Lanau et al., 2019). One substantial challenge is mapping material stock of buildings and infrastructure with high thematic detail and high spatial resolution, especially over longer time periods and across large areas (Lanau et al., 2019; Peled & Fishman, 2021). Such a representation of stocks is urgently needed, as the spatial distribution of stocks is highly variable between locations. At the same time, a temporal perspective on stocks is crucial to understand pathways for accumulation and development. Inflow‐driven modeling is able to assess material stock dynamics over time and across very large areas, for example, at the global to national level (Fishman et al., 2014; Wiedenhofer et al., 2019). However, its scale of analysis is strongly dependent on data availability, as it relies on statistical data on extraction, production, and trade, which is usually available at country level only. Spatially explicit, bottom‐up stocks accounting was primarily performed in local to regional case studies based on available cadastral or other reference building footprints, or OpenStreetMap data (Inostroza et al., 2019; Kleemann et al., 2017; Lanau et al., 2021; Miatto et al., 2019; Reyna & Chester, 2015; Tanikawa et al., 2015). If available, cadastral data are particularly suitable for stocks mapping because of their high spatial level of detail. However, they can either be expensive, difficult to acquire, or are inconsistently structured across administrative units (Biljecki et al., 2021). OpenStreetMap data are freely available, but its completeness is regionally inconsistent (Barrington‐Leigh & Millard‐Ball, 2017), and it is difficult to evaluate if they are up‐to‐date (Minghini & Frassinelli, 2019). So far, only a few mostly local/city‐focused studies mapped long‐term temporal dynamics of stocks, from historic building and infrastructure data (Guo et al., 2021; Merschroth et al., 2020; Miatto et al., 2021; Tanikawa & Hashimoto, 2010; Tanikawa et al., 2015).

Earth Observation (EO) data are increasingly used to map the built environment (Zhu et al., 2019). EO gathers information about the status of and processes on the Earth's surface from airborne or spaceborne sensors, and has previously been used for material stock mapping. For example, *Light Detection and Ranging* (LiDAR) data can create highly detailed 3D surface models. Although successfully used for a local material stock assessment (Schandl et al., 2020), LiDAR data are commonly acquired by flight campaigns, restricting LiDAR to regional acquisitions at high costs. Nighttime light data (NTL) were found to be a proxy for human activity and, thus, proportionally related to accumulated above‐ground stocks or to related variables, such as building volume (Liang et al., 2017; Peled & Fishman, 2021; Rauch, 2009; Takahashi et al., 2010; Vilaysouk et al., 2021; Yu et al., 2018). An advantage is their spatially consistent and global coverage with free access to historic archives (Elvidge et al., 2017). However, deriving stocks from NTL comes with technology‐inherent challenges. Particularly, the limited spectral and spatial resolution, that is, one spectral band of 250–1000 m spatial pixel resolution, hampers the identification of finer structures, different stocks types, or “dark” stocks (Elvidge et al., 2017; Peled & Fishman, 2021).

Optical and radar decameter resolution EO imagery has been widely established to map different variables of the built environment, for example, land cover composition (Li et al., 2018), building presence (Corbane et al., 2020), building height (Frantz et al., 2021), settlement structure (Demuzere et al., 2019), or derived parameters such as population (Stevens et al., 2015). This popularity is also due to three important EO systems, Sentinel‐1 (ESA Sentinel Online, 2021a), Sentinel‐2 (ESA Sentinel Online, 2021b) as well as Landsat (NASA, 2018). Those operational programs provide globally consistent and freely available data with a spatial resolution of 10 to 30 m, and Landsat provides the longest globally consistent time series of optical imagery since 1984. Recently, Haberl, Wiedenhofer et al. (2021) used spatially explicit data for the year ∼2018 on building cover area, building height, and building type derived from radar and optical Copernicus Sentinel‐1 and −2 (S1/S2) data as well as infrastructure data from OpenStreetMap in combination with stratified material intensity factors for a nation‐wide mapping of different materials and structures. They covered the whole of both Germany and Austria in a streamlined workflow at 10 m resolution. The Landsat data archive has not yet been exploited for historic high‐resolution material stock mapping.

The goal of this study was to map, quantify, and characterize the temporal dynamics of material stock patterns and population at a 30 m resolution for the entire territory of Germany from 1985 to 2018. For this purpose, the Landsat data archive and time series image analysis were utilized to track the annual addition (not accounting for removal or replacement) of material stock based on the high‐resolution maps of material stock for 2018 from Haberl, Wiedenhofer et al. (2021). Annual maps of gridded population at 30 m resolution are created following the workflow developed in Schug et al. (2021), based on mapping the annual addition of building volume from Frantz et al. (2021) to derive stocks per capita. We specifically addressed the following research objectives:

- Quantify spatial patterns of annual material stock addition in buildings and infrastructure across Germany from 1985 to 2018 using freely available Earth Observation data and existing recent material stock maps.
- Relate the development of material stock to population dynamics over time and identify, quantify, and interpret patterns of spatial–temporal development.

## METHODS

**FIGURE 1:**
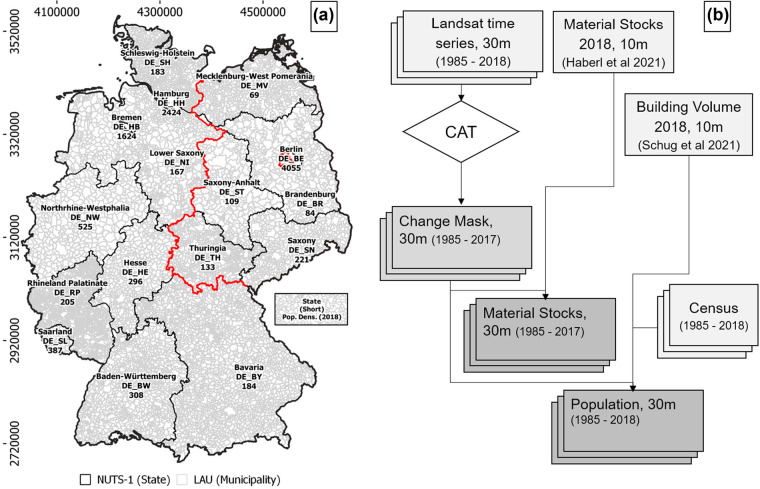
(a) Study Area Germany, its 16 federal states (NUTS‐1) and its 11,267 municipalities (LAU), including state abbreviations subsequently used in the manuscript. Red: Former Border between East and West Germany. (b) Workflow, creating historic Material Stock and Population maps from Landsat time series and previous data. For population density, see supporting information S1

Annual historic maps of material stock were calculated from 1985 to 2018 using the 2018 reference datasets in combination with yearly binary change masks from change‐aftereffect‐trend (CAT) analysis (Figure 1b). Going backward through time from 2017, the respective change mask was multiplied with all datasets from the following year to obtain stocks in that year. This means that we retrospectively calculated the annual addition of material stocks from a recent material stocks map. For example, stock maps for 2017 were derived by a multiplication of the 2018 stocks maps with the 2017 binary change mask. These change masks were also used to create annual gridded population maps. Gridded population maps represent the number of people per grid cell, that is, per pixel. We first created maps of the addition of residential building volume for each year based on a volume map for 2018. We then used annual census data and redistributed total population counts to pixels using a dasymetric mapping approach (Leyk et al., 2019). Time series data of material stock and population served as a basis for the analysis.

### Study area and census data

Germany covers an area of about 357,000 km^2^, had about 82.79 million inhabitants in 2018 and is structured into 16 federal states (BKG, 2019; Figure 1a). There were three reasons for choosing this study area. First, high‐resolution maps of material stock as well as buildings and infrastructure area, building height, and type were available from previous works (Frantz et al., 2021; Haberl, Wiedenhofer et al., 2021; Schug et al., 2020, 2021). Second, settlement structure in the study area is diverse and contains urban agglomerations, rural and suburban areas and large gradients in‐between, with population density ranging from 69 to 4055 km^−2^ in the federal states (BKG, 2019). Third, Germany is a promising thematic case for a proof of concept due to historic events. The Reunification of the former Federal Republic of Germany (former West) and the German Democratic Republic (former East), for example, was a process that began in 1989, and had a number of long‐lasting effects on migration (Glorius, 2010), economic modernization, and restructuring (Burda & Hunt, 2001). This study used annual census data from Eurostat NUTS‐1 (*Nomenclature des unités territoriales statistiques*) units and aggregates results on LAU (*Local Administrative Unit*) administrative units (Eurostat, 2016). Census data from 1990 to 2018 as well as data from 1985 to 1990 for all former states of the German Federal Republic originated from the German Federal Statistical Office (Federal Stat. Office, 2021). Census data from 1985 to 1990 for all former districts of the German Democratic Republic originated from the Statistical Office of the German Democratic Republic (digitized in DZ, 2021). All results are presented in today's administrative boundaries.

### Reference maps from previous works

We started from spatially explicit high‐resolution material stock maps for 2018 created in Haberl, Wiedenhofer et al. (2021). These represented the mass of stocks for 13 different materials grouped into non‐metallic minerals, metals, biomass‐based and fossil‐fuels‐based materials, as well as 26 different building and infrastructure types. Those layers had been generated by combining information on building and infrastructure area (Schug et al., 2020), building height (Frantz et al., 2021), and building types (Schug et al., 2021), all based on S1/S2 data with a spatial resolution of 10 m representing the physical setup of the built environment. Building area [m^2^] and height [m] were used to derive the volume of buildings [m^3^]. In order to translate mapped building volume [m^3^] and infrastructure area [m^2^] into weight, individual material intensity (MI) factors [t/m^2^ or t/m^3^] were derived from the literature and specifically applied for each building and infrastructure type. While the original data contained information on seven material types (e.g., concrete, metals, bricks), here we used the sum of all material types only. For further information we refer to Haberl, Wiedenhofer et al. (2021). For this study, we focused on the following aggregated above‐ground stock layers:

• Total material stock…
  • … in buildings
    • … in commercial and industrial buildings
    • … in multi‐family residential buildings
    • … in single‐family residential buildings
  • … in infrastructure
    • … in road infrastructure
    • … in rail infrastructure
    • … in other infrastructure

We refer to Schug et al. (2021) for further information about the used building volume layer. For consistency with the spatial resolution of Landsat, all datasets were aggregated to a resolution of 30 m.

### Change‐aftereffect‐trend analysis with Landsat time series data

We downloaded all available optical Landsat imagery for our study area from 1985 to 2020, acquired from TM, ETM+, and OLI sensors (Collection 2, Level 1; USGS, 2021) using *landsatlinks* (Ernst, 2021), with a cloud coverage of less than 70%. All images were pre‐processed through the Framework for Operational Radiometric Correction for Environmental monitoring (FORCE; Frantz, 2019). This included, among others, cloud and cloud shadow masking, radiometric correction, re‐projection into a common coordinate system, and creating data cubes (Frantz, 2019). Pre‐processing is necessary to establish comparability across images from different sensors and through time and space.

**FIGURE 2:**
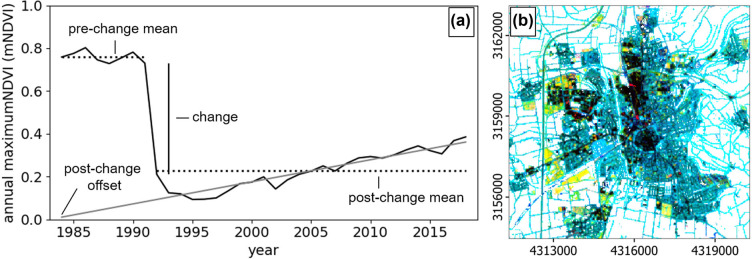
(a) Components of change‐aftereffect‐trend analysis. Data is example data. (b) Exemplified spatial representation of change (red, 0–0.25), pre‐change mean mNDVI (green, 0.2–0.8) and post‐change mean mNDVI (blue, 0.2–0.8). Dark areas represent little change with little vegetation. Blue/turquoise areas represent little change with stable or increasing vegetation. Red areas represent high change with a lower pre‐change mean than post‐change mean (vegetation re‐development). Yellow areas represent high change with a higher pre‐change mean than post‐change mean (building activity). The year of change is not visualized in (b) and can be different for each pixel. For data, see 10.5281/zenodo.6909185

We performed a CAT analysis for the whole study area as implemented in FORCE. CAT is a method for multi‐temporal change detection, suggested by Hird et al. (2016), and captures gradual (*trend*) or abrupt (*change*) non‐seasonal landscape changes based on a multi‐annual time series of a spectral vegetation index. CAT divides this time series into two sections, pre‐ and post‐change, based on the detected year of abrupt change, assuming that only one single change occurred during the study period (Figure 2). We assumed that the complete removal of vegetation in Germany is an indicator for building activity, and that change was discrete, that is, from zero to the material stock or building volume observed in the reference map of 2018. The CAT analysis was performed on an annual basis, where individual satellite acquisitions are aggregated to yearly measurements by using the maximum value of the Normalized Difference Vegetation Index (NDVI; Tucker, 1979) per year. The NDVI is a robust indicator for the peak of the growing season, ignoring intra‐annual seasonal vegetation cycles, and ranging from −1 to 1, with 1 being the maximum vegetation. Thus, the year of change is determined based on an abrupt decrease in maximum NDVI (mNDVI). We considered the *mNDVI* of all observations within a year between day 70 and day 304 to avoid data artefacts resulting from snow cover in winter. While Hird et al. (2016) suggested to detect the year of change based on the maximum interannual absolute difference of the index within subsequent years, here we created a change score *s* for each year *y* that only detects negative change (i.e., vegetation removal) and that favors persistent change (i.e., longer than 2 years, Equation 1). This increased the robustness against data‐sparse years or years with heavy cloud coverage and avoided a potential misinterpretation of change processes.

1 $$ {s}_{y}={\rm{mNDVI}}_{y-1}-{\rm{mNDVI}}_{y}\ensuremath{\times{}}\left({\rm{mNDVI}}_{y-1}-{\rm{mNDVI}}_{y+1}\right) $$

CAT assigned a year and an amplitude of change, as well as additional change metrics of the annual mNDVI to each 30 × 30 m pixel. As CAT detects change in each and every pixel based on the highest change score, a set of criteria is required to distinguish actual change from seeming change. We only considered changes in pixels that satisfy the following four criteria:

1. a minimum difference of 0.25 in mNDVI between the detected year of change and the previous year,
2. a post‐change mean of 0.6 or smaller, that is, an area mostly non‐vegetated after the change,
3. a pre‐change mean larger than the post‐change mean, excluding areas with strong vegetation regrowth suggesting a non‐permanent change, or data artefacts,
4. a pre‐change mean larger than the post‐change trend offset multiplied with 1.2, which eliminated areas with sudden regrowth and subsequent ongoing vegetation decline after the change, suggesting transient change only (Figure 2).

### Validating estimated changes

We validated the annual change maps using a two‐step stratified random sampling strategy, as suggested in Olofsson et al. (2014). Sampling was stratified by the mapped area shares of the six sub‐categories—area of single‐family, multi‐family, and commercial/industrial buildings as well as area of road, rail, and other infrastructure. In a **first step**, we assessed the performance of the CAT approach in detecting change, that is, distinguishing *change* and *no‐change* in the six thematic classes (i.e., a 12‐class stratification; Table Table 1). In a **second step**, we assessed the performance of CAT to detect a specific year of change. Here, eight temporal classes were formed and combined with the six thematic classes, resulting in a 48‐class stratification (Table Table 1). Temporally invariant surfaces were not part of this step. According to Olofsson et al. (2014), the number of samples is dependent on the mapped area proportion of each class *W*_*i*_, a target user's accuracy *U*_*i*_ and a target standard error *S* for the estimated overall accuracy (Equation 2), simplified for large populations). The samples were allocated to the strata based on their proportion of mapped area.

2 $$n = {\left( {\frac{{\sum {\left( {{W}_i \times \sqrt {{U}_i \times \left( {1 - {U}_i} \right)} } \right)} }}{S}} \right)}^2$$

**TABLE 1:** Number of samples for both steps of the validation approach in all thematic and temporal change and no‐change classes

		Total number of samples					
		Buildings			Infrastructure		
		SF	MF	CI	RD	RL	OT
Step 1	Change	58	18	118	61	5	16
	No change	577	168	270	811	105	46
Step 2 Change from … to …	1986–1989	30	12	60	30	11	8
	1990–1993	52	15	103	48	16	13
	1994–1997	66	16	89	18	60	13
	1998–2001	65	18	102	72	20	15
	2002–2005	38	10	59	53	15	12
	2006–2009	30	13	84	39	17	13
	2010–2013	29	12	89	32	13	9
	2014–2018	23	10	88	22	8	6

Abbreviations: CI, commercial/industrial buildings; MF, multi‐family buildings; OT, other infrastructure; RD, roads; RL, rails; SF, singlefamily buildings.

Only pixels with a building area share of >25% and an infrastructure area share of >33% per pixel qualified as a validation sample. This avoided a selection of pixels covered by both surface types with no clear major class. Because of their small surface area, rail and other infrastructures were merged after stratification.

We collected reference data on change/no‐change and the year of change for all samples of the first and second steps. This information was manually collected directly from the satellite imagery based on visual interpretation. This was supported by two datasets, provided as image chips: (1) the time series of annual mNDVI values as used for the CAT analysis, and (2) the reflectance spectrum of the Landsat acquisition for which the mNDVI was calculated within each year. The first allowed for a plausibility check of the year‐of‐change detection. The second provided spatial and temporal context on the surface and its surroundings. Change was labeled if vegetation was permanently replaced by an impervious structure, indicated by no, little, or very slow increase of the mNDVI after sudden vegetation removal. The validation did not assess the quality of the underlying strata, as the quality of building types and infrastructure area were reported previously (Frantz et al., 2021; Haberl, Wiedenhofer et al., 2021; Schug et al., 2021). Locations with a high labeling uncertainty were re‐assessed by another interpreter.

## RESULTS

### Validation of change maps

After labeling, 2.5% of the samples were manually excluded, as they were erroneously placed on permanently non‐built‐up surfaces. These errors originated from the reference material stocks map for 2018 and cannot be ascribed to the change analysis. In the first step of the validation strategy, 92.1% of all change samples and 95.3% of all no‐change samples were correctly classified. Regarding step two, the share of correctly detected change years ranges from 85.1% for changes between 1998 and 2001, and 95.3% for changes between 1994 and 1997. For samples where a specific year of change was detected, the offset between the detected and the actual year of change was lower than 1 year.

### Material stock development and the German reunification

**FIGURE 3:**
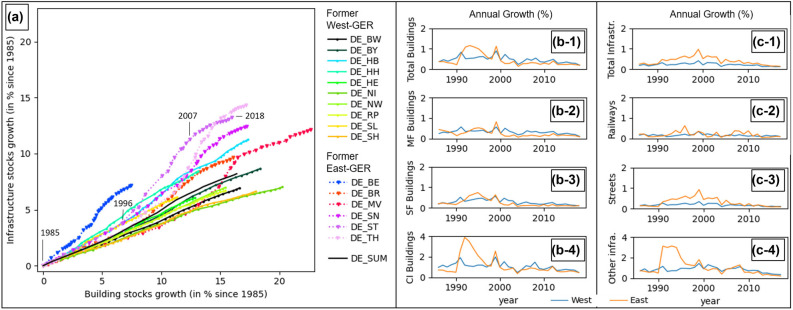
(a) Cumulative building and infrastructure stocks growth per state (including national sum) for each year until 2018 (base year 1985). One point = one year. See Figure 1 for state locations. Top: States in former West Germany, Center: States in former East Germany. (b) Annual growth rates (in percent) of stocks in buildings, and (c) in infrastructure as well as sub‐categories in Western (blue) and Eastern (orange) states from 1986 to 2018. CI, commercial/industrial buildings; MF, multi‐family housing; SF, single‐family housing . No growth rates provided for base year 1985. German reunification in 1990. For data, see supporting information S1

**FIGURE 4:**
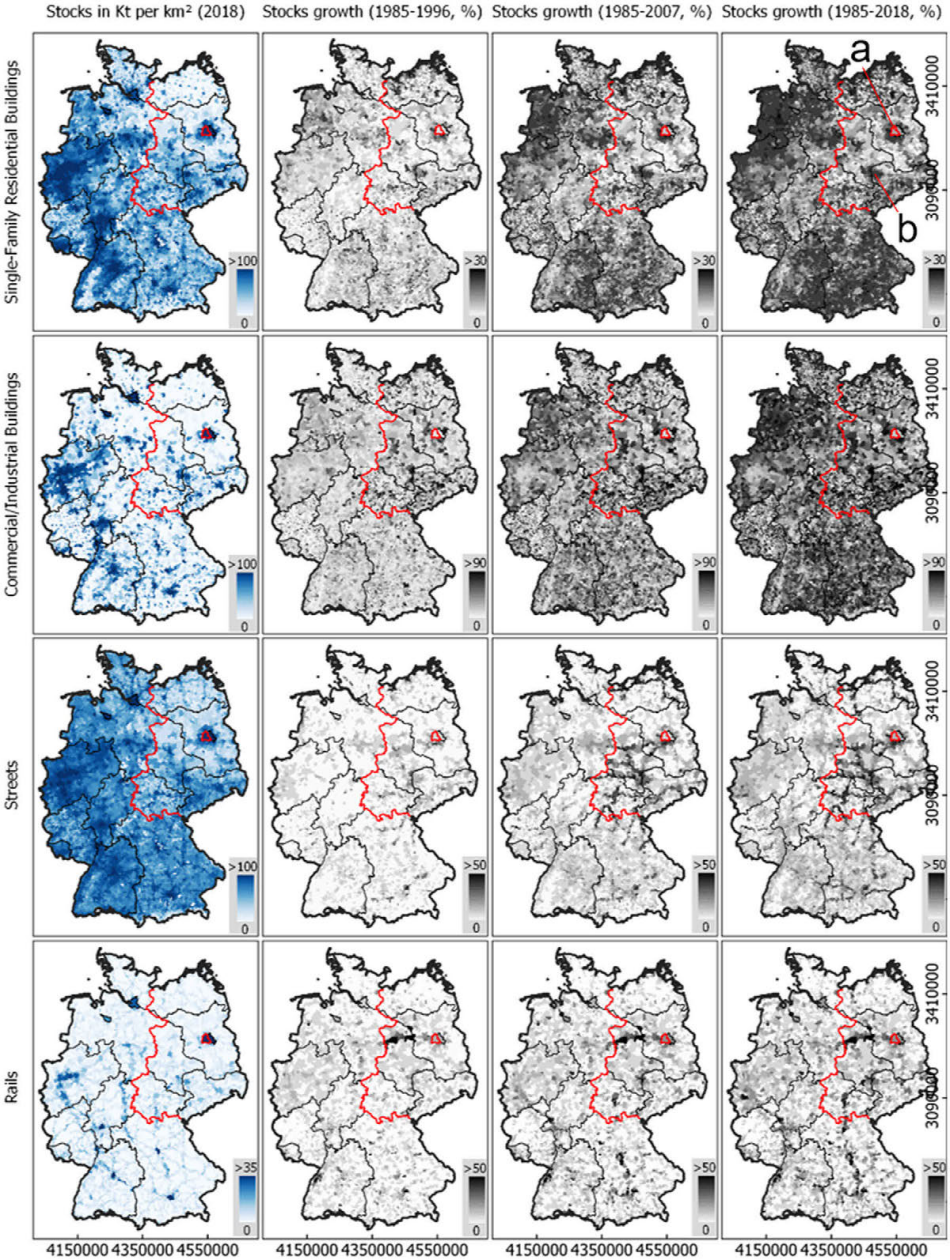
Material stock density (kt/km^2^) in 2018 and growth (%) in three selected periods in Germany in four example categories (single‐family buildings, commercial and industrial buildings, roads, and rails). Data for 2018 from Haberl, Wiedenhofer et al. (2021). All other years from this work. Red: Former Border between East and West Germany. Regular time steps of 11 years. German reunification in 1990. For data, see supporting information S2 and S3

Total material stock in Germany grew from 31.0 Gt in 1985 to 35.1 Gt in 2018 (+ ∼13%). Building stocks grew from 18.8 to 21.9 Gt (+ ∼17%), and infrastructure stocks from 12.2 to 13.2 Gt (+ ∼8%, Figure 3a, *DE_SUM*). National annual stock growth is rather steady, with an average annual growth rate of 0.5% for total stock, 0.5% for building stock and 0.2% for infrastructure stock. At state level, stock growth is highly dynamic and differentiated. The growth of building stock during the whole period ranges from 7% in Berlin (DE_BE) to ∼23% in Mecklenburg‐West Pomerania (DE_MV), and the growth of infrastructure stocks ranges from 6% in Hesse (DE_HE) to 14% in Thuringia (DE_TH). Three major types of development stand out. First, among the 16 states, the city states Berlin and Hamburg as well as Saarland and Hesse (DE_BE, DE_HH, DE_HE, DE_SL) have a considerably lower growth of building (7% to 13%) and infrastructure stock (6% to 8%) than the German average. Second, large states in Western Germany experienced a steady and spatially homogeneous growth of both buildings (15% to 20%) and infrastructure (6% to 8%). Third, states in Eastern Germany have a particularly high growth of infrastructure, with an acceleration after the reunification in 1990 until the late 2000s and a subsequent deceleration. While the growth of buildings and infrastructure as well as in their sub‐categories is on a constantly low level in Western states, annual growth rates were higher in Eastern states (Figure 3). Here, annual building growth rates were twice as high as in Western states in the mid‐1990s (Figure 3b‐1). Particularly, the growth of single‐family buildings is constantly higher (Figure 3b‐3), and commercial and industrial building stock (Figure 3b‐4) grew with up to 4% per year. With regard to infrastructure (Figure 3c‐1), annual growth in Eastern states was consistently higher compared to Western states after 1990, with a constantly higher rate for roads (Figure 3c‐3) and a massive growth of other infrastructure such as parking lots right after 1990 (Figure 3c‐4).

### Spatial stock development patterns

From 1985 to 2018, material stock in different building and infrastructure types grew at different rates. Stocks in single‐family buildings grew from 10.4 to 11.4 Gt (+ ∼9%), in multi‐family buildings from 4.2 to 4.6 Gt (+ ∼11%), in commercial and industrial buildings from 3.9 to 5.8 Gt (+ ∼42%), in road infrastructure from 11.0 to 11.7 Gt (+ ∼7%), in rail infrastructure from 0.42 to 0.44 Gt (+ ∼6%), and in other infrastructure from 0.7 to 0.9 Gt (+ ∼32%).

Figure 4 illustrates the density and relative development of stocks in municipalities in four example categories. Across the categories, patterns of stock distribution resemble and are particularly concentrated in and around major agglomerations. North‐East Germany features overall lower stock quantities. The patterns of commercial and industrial building stocks are more granular, with more abrupt transitions, than those of single‐family buildings. Road stocks reveal an underlying structure of inter‐city networks in otherwise rural areas. Rail stocks accumulated along axes between agglomerations. Since 1985, there was considerable development in all categories (Figure 4). Single‐family building stocks grew across Southern and North‐Western Germany. Additionally, growth occurred in and around densely populated places (Figure 4). Commercial and industrial building development was regionally more balanced, with particular hotspots in all parts of the country. Development was stronger in Eastern Germany in the first period, around and after the reunification in 1990. Road stocks grew along major axes, particularly in Eastern Germany until the mid‐2000s.

### Population

**FIGURE 5:**
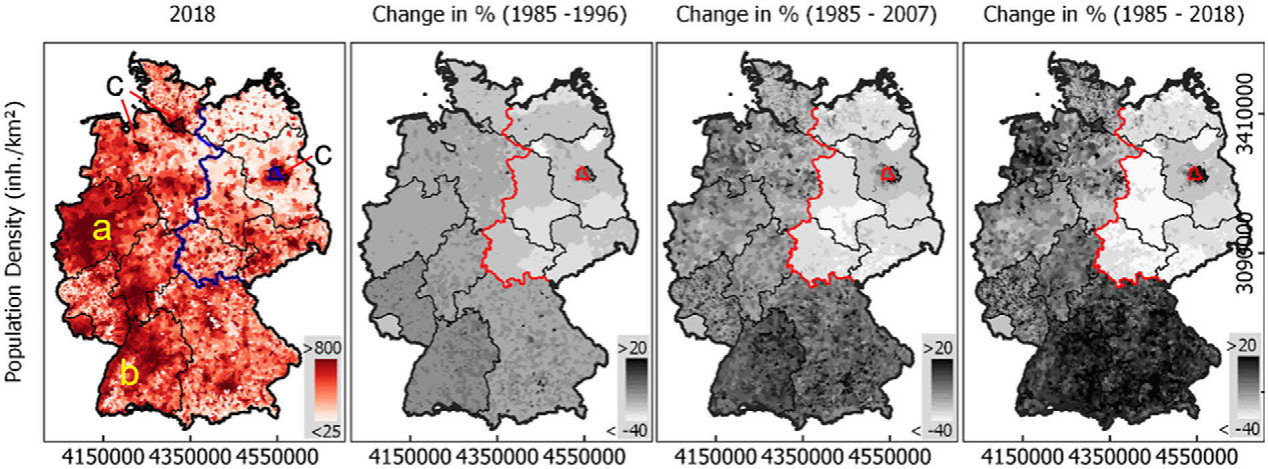
Population density (cap/km^2^) in 2018 and population change (%) in three selected periods in German municipalities. Data for 2018 from Schug et al. (2021), all other years from this work. Blue/Red: Former Border between East and West Germany. Regular time steps of 11 years. German reunification in 1990. For data, see supporting information S2 and S3

Total population in Germany grew from 79.5 million in 1985 to 83.2 million in 2018 (Federal Stat. Office, 2021). Population across Southern and Western federal states grew between 11.9% (DE_RP) and 18.6% (DE_BW), with a growth of 19.2% in the formerly divided capital Berlin (DE_BE). In states of former East Germany, population decreased between 6.7% (DE_BB) and 27.3% (DE_ST), including a fairly sharp drop between 1990 and 1993 and a subsequent constant decline. Population density in 2018 was highest in and around major agglomerations, such as the Ruhr District (Figure 5a), along the Rhine valley (Figure 5b) and the three city states (Figure 5c). North‐East Germany has an overall lower population density. Interestingly, overall population growth did not primarily occur where population density was high already (Pearson's *r* = 0.01). While growth or decline were spatially rather homogeneous in some states, others experienced a spatially more unbalanced development (e.g., DE_NI, DE_RP). In Brandenburg (DE_BB), for example, growth focused on the periphery of Berlin, DE_BE).

### Material stock and population

Material stock and population are generally correlated on a municipal level. We find that, over the entire 33 years, population density strongly correlates with a high building‐to‐infrastructure stock ratio (from 1985 to 2018, all municipalities, Pearson's *r* = 0.82, *R*^2^ = 0.75), indicating a higher share of buildings compared to infrastructure stock in densely populated areas. Population density is also strongly correlated with a high multi‐family‐to‐single‐family building stock ratio (Pearson's *r* = 0.80, *R*^2^ = 0.65), indicating that higher population density can be found in areas with more multi‐family compared to single‐family buildings. Interestingly however, the growth of building and infrastructure material stock is not correlated to population density (Pearson's *r* = 0.10, *R*^2^ = 0.0063), indicating that stock growth primarily occurs in areas with lower population density, a phenomenon often discussed as “urban sprawl.”

**FIGURE 6:**
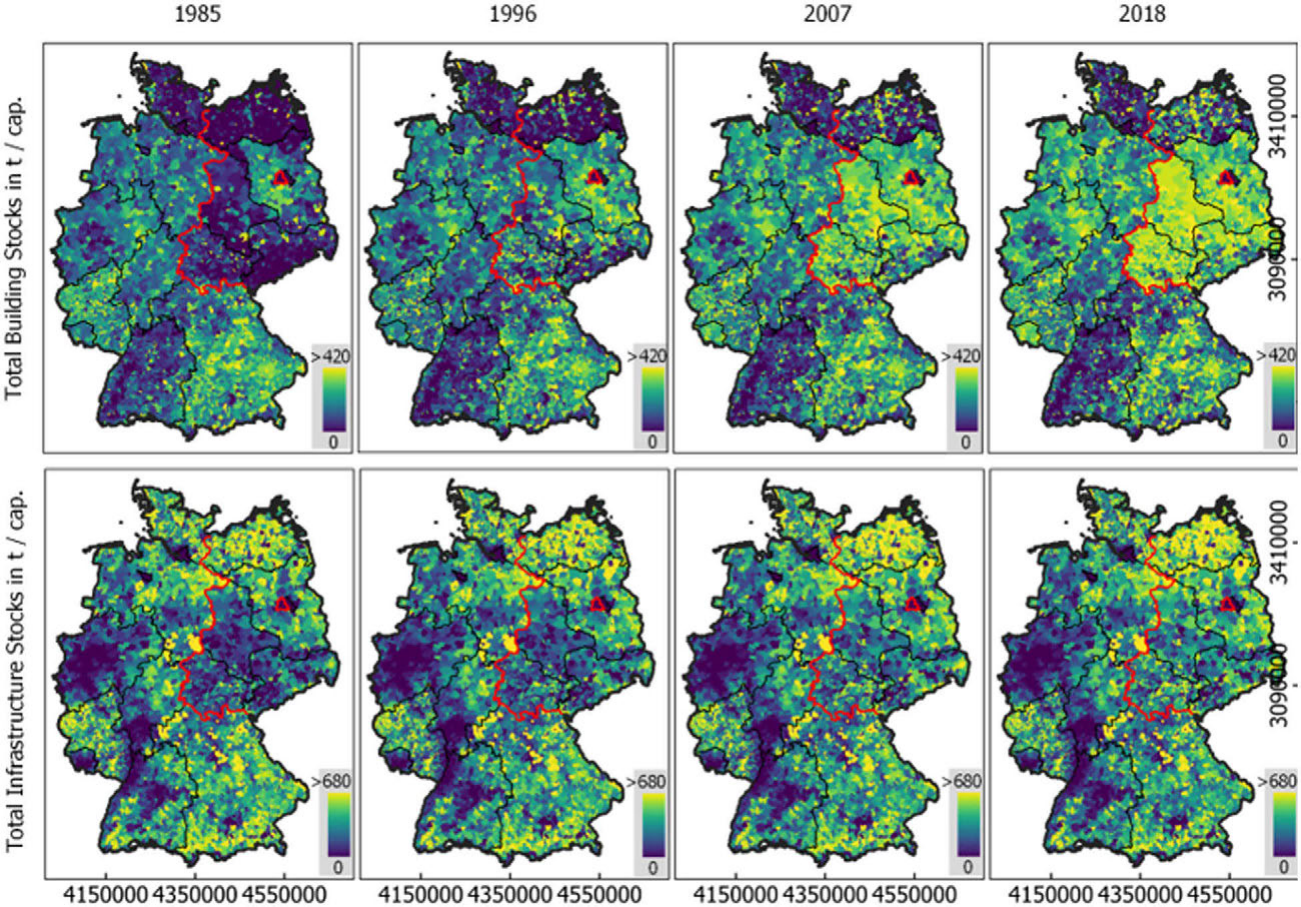
Material stock per capita in buildings (top) and infrastructure (bottom) per municipality in four different years. Red: Former Border between East and West Germany. Regular time steps of 11 years. German reunification in 1990. For data, see supporting information S2 and S3

Material stock per capita is highly variable on a local level (Figure 6). While there is important growth of stocks per capita in Eastern Germany (e.g., building stocks in Thuringia, DE_TH, of 217 t/cap in 1985 and 322 t/cap in 2018), development is rather slow in Southern and Western states. Some feature clear gradients, particularly expressed in infrastructure per capita, with lower stock in urban and higher stock in rural areas (e.g., Baden‐Wurttemberg, DE_BW, Hesse, DE_HE). Densely settled regions show little development (e.g., Berlin, DE_BE, infrastructure stock of 32 t/cap in 1985 and 28 t/cap in 2018).

**FIGURE 7:**
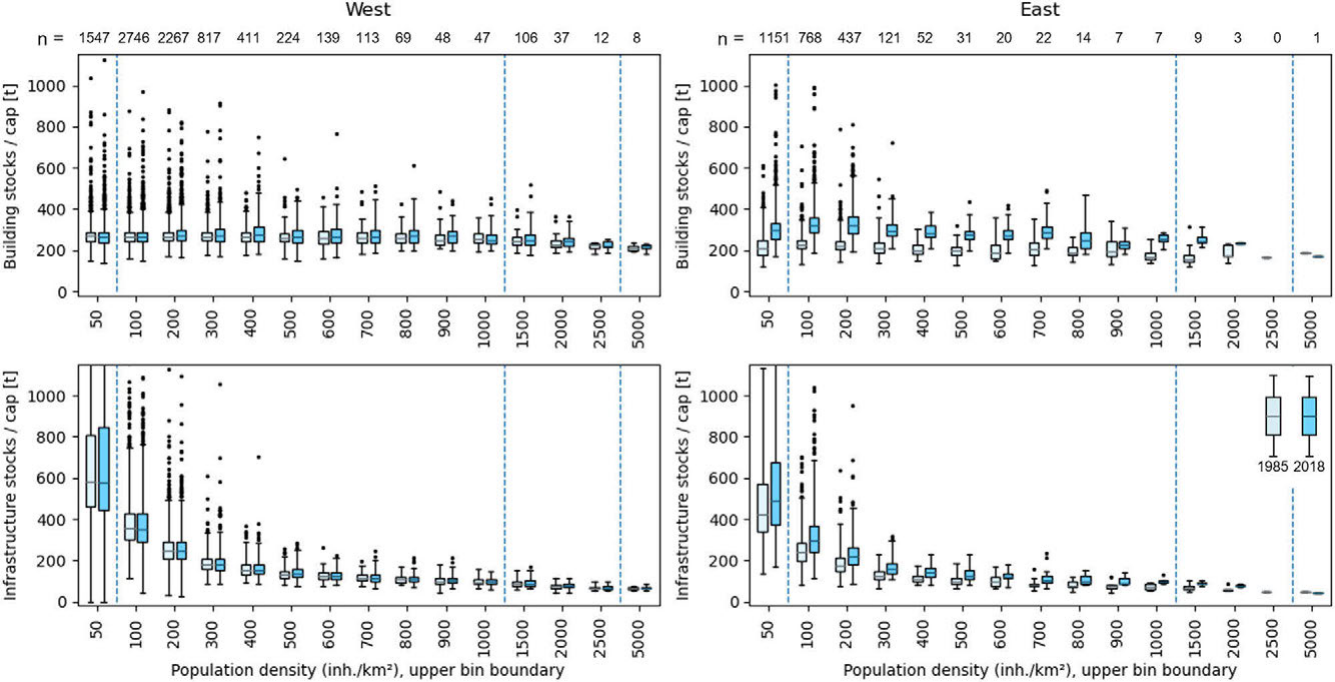
Building (top) and infrastructure (bottom) stocks per capita in Western (left) and Eastern (right) federal states. Boxplots represent stock values of all municipalities in 1985 and 2018 (boxplot color). Population density (*x*‐axis) represents the upper boundary of the boxplot bins, for example, 500 for all municipalities with a population density between 400 and 500. *x*‐axis not to scale below 100 and above 1000 (scale changes divided by dashed blue vertical lines). *n* refers to the number of municipalities per bin in 2018. *n* for 1985 might differ. For data, see supporting information S3 and S4

Figure 7 shows the development of building and infrastructure stock per capita in all municipalities in Western and Eastern federal states in 1985 and 2018 for population density classes. While infrastructure stocks per capita are strongly related to population density (i.e., higher stocks per capita in municipalities with lower population density) in both Western and Eastern states, this relation is less pronounced for buildings. The lowest observed stocks per capita across all municipalities in 2018 (>300 inhabitants/km^2^) are ∼180 t/cap for buildings and ∼150 t/cap for infrastructure. Stocks per capita in both categories are temporally stable in Western states with only slight changes in median values. However, stocks per capita are increasing in Eastern states, resulting in higher building stocks per capita compared to Western states in 2018, and converging infrastructure stocks per capita.

## DISCUSSION

### Methodology

Our modified CAT transform is a robust and straightforward approach to create annual change masks. It requires comparatively little data, particularly when using the mNDVI, as only one annual observation during peak vegetation is needed. The accuracy of the change/no‐change as well as the year‐of‐change detection is very good, and comparable to the results presented in Hird et al. (2016) for mapping urban expansion in Canada. Validation followed an established scheme, but with an adapted approach to facilitate data handling. A two‐step validation allowed for a manageable procedure with a moderate number of samples, while a single‐step approach would have resulted in an enormous number of points when respecting the requirements for an area‐proportional sampling. This is also why we conflated years into groups, which also seems sensible because building and infrastructure stocks change relatively slowly.

#### Challenges and limitations

The presented approach can be challenged regarding the input data and the logic of the CAT analysis. First, the quality of historic mapping results is directly linked to the quality of underlying maps used to create the initial material stock and building volume map of 2018. Both rely on an accurate representation of building area and height. While Schug et al. (2020) and Frantz et al. (2021) report only moderate errors here, they can accumulate upon multiplication. Material stock inputs are additionally driven by the applied material intensity factors, which are subject to high uncertainty, as samples to derive them are very limited. Haberl, Wiedenhofer et al. (2021) reported low to moderate errors in the stock maps for 2018, albeit with potential overestimations compared to other studies, but reference data to evaluate this outcome are sparse. Material intensity factors are also used invariant in time, as information on building age is missing in this approach. Furthermore, this work used state‐level census data to map gridded population for all years. The quality of population grids was affected by census inconsistencies. For example, German census data were adjusted in 2011, resulting in sudden population drops in almost all states because population statistics were only propagated from the 1986 census in West Germany and the 1995 building census in East Germany (Federal Stat. Office, 2019) until 2010, showing a relevant difference to actual demographics. Additionally, population statistics in former East Germany had been subject to manipulation before 1990 (Lippe, 1996). Second, the fact that the CAT analysis as we applied it can only detect one single change in the time series introduces uncertainties where the built environment was more dynamic. For example, our approach did not account for the common removal and replacement of stocks, nor the shift between stock types, which occurred before the year 2018. That affects areas where buildings or infrastructure were removed and rebuilt without changing its extent, but its shape, height, structure, or function. Accounting for net removals and shifts between stock types would require information about historic built‐up types, instead of maps that are based on net accumulation and change until a given baseline year. In our study, material stock replacement and removal could have an impact on the results across the whole area, but particularly in East Germany, where building stock was comprehensively modernized after the reunification, or demolished after population decline caused by de‐industrialization. Additionally, the analysis did not account for underground infrastructure.

#### Further Research

As this workflow used data derived from freely available and globally consistent data, we consider it to be transferable to environments around the world, provided that the link between vegetation removal and building activity is given. It can also be used with any other current and future spatially explicit material stock or population map, also at lower spatial resolution. The CAT approach requires a careful consideration of possible historic data gaps if only few acquisitions, or acquisitions distant from the peak vegetation, are available in the time series. In principle, one data acquisition after the removal of vegetation in the respective year would suffice to detect change.

For an accurate understanding of the relationship between population and stocks, a high temporal density of census data is desirable. Further refinements to underlying material stock data in the future, for example, by also integrating material intensity factors by age class, could make this approach an independent alternative to inflow‐driven stocks modeling over time. Further research could explore how well such stock estimates could be used to derive spatially explicit estimates of material flows from construction, maintenance, and demolition. This workflow can also contribute to future Long‐Term Socio‐Ecological Research (LTSER; Singh et al., 2013), as it helps downscaling the SEM to the pixel level. Moreover, it offers the possibility to quantify the emergence of resource‐gobbling patterns, for example, as a basis to analyze changes in social practices (Haberl, Schmid et al., 2021) in response to infrastructure changes, as well as the emergence of risk‐prone infrastructures, for example, in Alpine regions.

### Material stock and population

Results are affected by the quality of the change analysis, uncertainties in the validation procedure, the quality of the initial material stock map for 2018 (quality reported in Haberl, Wiedenhofer et al., 2021), the quality of the adopted method for population mapping (potential errors reported in Schug et al., 2021), and other assumptions. With 0.5% per year, the growth of material stock reported in this study is on an overall lower level compared to inflow‐driven studies for other industrial countries (e.g., 0.8% to 1% in the 2000s in the United Kingdom, Streeck et al., 2020, or 1.2% after 2010 in the *industrial old world*; Wiedenhofer et al., 2021). While reference data on commercial and industrial buildings are not available, we compared residential building stocks to official building statistics (Deutsches Statistisches Bundesamt, 2021). The latter report an increase of dwellings of ∼27% in Germany between 1987 and 2018, which is considerably more than the ∼10% growth of single‐ and multi‐family buildings reported in this study. Knowing that both are not perfectly comparable because of different units of measurement, potential reasons for this discrepancy are that in contrast to our study, official statistics record building type conversion and replacement, densification, or extensions and adding floors to existing buildings. Additionally, the building type classification used in this study tends to classify inner‐city mixed‐use buildings as commercial buildings, even though they could have a relevant residential function. Being the first of its kind, this work provides first estimates for an encompassing independent approach to material stock mapping. However, we underline that further research will be required to understand the impact of uncertainties within the used data and methods to dynamic material stock monitoring. We are confident that possible inaccuracies in total material stock development do not affect spatial patterns.

#### The urban–rural gradient

Material stock and population distribution and dynamics show distinct regional patterns. Although they are concentrated in and around large cities, both do not grow most where population density is high. While population growth can be observed in all landscapes, building stocks grew stronger in suburban and rural areas than in urban centers. Commercial and industrial building stocks do not seem to follow such a spatial–temporal pattern. Patterns are slightly different for mobility infrastructure, where growth is represented by network structures connecting major agglomerations. Results suggest that material stock growth in Germany is primarily represented by sprawl, and not by an ongoing spatial concentration or densification of stocks. Acknowledging the lack of stock replacement or vertical building growth in our study, this statement would, however, require verification in further research. Results also suggests that there is a minimum quantity of stocks per capita required for humans living in a highly industrialized society like Germany, as no municipality has a building stock below ∼180 t/cap. This also applies to infrastructure stocks, but only above a population density of 300 cap/km^2^.

### East and West Germany

While material stock per capita in Eastern federal states of Germany are considerably lower in 1985 (Figures 6 and 7), we found evidence for important catch‐up effects after the reunification compared to Western federal states. This is represented by higher growth rates in nearly all stocks types (Figure 3). Building stocks and other infrastructure grew rapidly in the 1990s, after the reunification, and a high‐resolution map shows that this was partly caused by the construction of many suburban mall‐like commercial structures and modern industrial parks, present in West Germany decades earlier. The increase of stocks per capita in Eastern states after 1990 compared to Western states might be caused by three overlapping developments: (1) the higher growth of single‐family housing, (2) the massive growth of commercial and industrial buildings, and (3) the population decline. While this could be interpreted as Eastern municipalities becoming less stock efficient over time, we have to note that Eastern Germany is suffering from substantial migration, leaving buildings and infrastructure abandoned and under‐used, which is characteristic for shrinking societies and indicates large potentials but also substantial limitations for recycling and a more circular built environment (Schiller et al., 2017).

## CONCLUSION

This study provides annual data on material stock and population dynamics based on consistently available Earth Observation data. It presents a workflow for a wall‐to‐wall approach that maps the development of different stock types at high spatial resolution. Material stock and population patterns and dynamics in Germany follow both an urban–rural gradient and an East–West divide. It seems that there is a minimum amount of building and infrastructure stock required, and that growth of material stock primarily occurs because of sprawl. The German reunification in 1990 had a major impact on population and stock development, specifically in East Germany. We consider this approach promising to bridge the gap between spatially explicit, but temporally static high‐resolution stocks‐driven maps and inflow‐driven material stock modeling that is temporally dynamic, but bound to administrative units providing statistics. Compared to nighttime light approaches, it provides detail on stocks types and their relation to population. The approach is in principle transferable to other world regions, and its workflow allows the modular implementation of additional or alternative datasets, if available. As a proof‐of‐concept study, this research requires further insights into map uncertainties and local quality variations, but contributes to an advanced understanding of the high‐resolution stocks‐flow‐service nexus. It offers an alternative to established modeling approaches with a novel high‐resolution historic dimension.

## Supplementary Information

**Supporting Information S1**: This supporting information provides underlying data of Figure 3 (.xls), i.e. relative growth of material stock in buildings in infrastructure, annual and compared to the base year 1985, for all federal states and state groups.

**Supporting Information S2**: This supporting information provides underlying data of Figures 4 to 7. It contains material stock quantities for all building and infrastructure types [in t] as well as population for all German municipalities (boundaries of 2018) for every year from 1985 to 2018. This data is provided as an ESRI Shapefile, ready to be imported into any GIS software.

**Supporting Information S3**: This supporting information provides underlying data of Figures 4 to 7. It contains material stock quantities for all building and infrastructure types [in t] as well as population for all German municipalities (boundaries of 2018) for every year from 1985 to 2018. This data is provided as a comma separated file.

**Supporting Information S4**: This supporting information provides underlying data of Figures 4 to 7. It contains material stock quantities for all building and infrastructure types [in t] as well as population for all German municipalities (boundaries of 2018) for every year from 1985 to 2018. This data is provided as a multi‐sheet Excel file.

## Data Availability

The data that support the findings of this study on a municipality level are made available in the supporting information. The raster datasets at 30 m spatial resolution for all years and stock types as well as population are accessible at 10.5281/zenodo.6909185.
